# Supplementary material for: Genetic analysis of cis-enhancers associated with bone mineral density and periodontitis in the gene SOST
Source: PLoS One. 2025 Mar 24;20(3):e0319259. doi: 10.1371/journal.pone.0319259 (PMC11932464; doi:10.1371/journal.pone.0319259)
Supplement: S1 File — Legend: This S1 File contains Appendix Material, Appendix Figures 1-3, and Appendix Tables 1-5 that provide additional data and analyses relevant to the main manuscript. (DOCX) [file pone.0319259.s001.docx]

**Appendix**

**Appendix Material**

**Bioinformatic analysis of associated SNPs predicts allele-specific effects on TF CEBPB binding affinities in functional cis-regulatory elements**

**ChIP-Seq peak 1**

From: Transcription Factor ChIP-seq Clusters (338 factors, 130 cell types) from ENCODE 3

Factor: CEBPB

Position: chr17:41810335-41810590 (hg19/Human)

Genomic Size: 256

cell type:

**A549**: Cluster Score Signal (out of 1000) = 337.00

**IMR-90**: Cluster Score Signal (out of 1000) = 1000.00

>hg19_dna range=chr17:41810299-41810590 5'pad=0 3'pad=0 **strand=-** repeatMasking=none

GTCACCTTGTTAGTATAAACTCAGGCATGGTCCCAGTGGCTCACTATGAA

TAACAAAGACATTCCTATCACTTGGGAAATTCCAAGGGTTTAGAAGCTAC

CTCTCAGAAACCCAAGACAACTGGAGAAATTATTATGCAACAAATGCCAG

CCTATCTTCACCACAGTGTGCCCCATGACCCCTTTTCTATGCTCTTGGCT

TAGGTCAGGCAGAGCAGCTTGCGGCTCTCCATGTGGACCTGGAGATTTTT

CACCTCGCTTCCTTTGCTTGCACTGAT[C/T]CCTTTGTCTGGAAT

CEBPB binding motive

GFI binding motive

distance 195 bp

This table shows extracted p-values, Matrix IDs, Matrix names, and calculated 'corrected P_values' from the DNA sequence of **ChIP-Seq peak 1**. It is sorted form the lowest to the highest p-value.

| Rank | P-value | Matrix_ID | Matrix_name |
| --- | --- | --- | --- |
| 1 | 0,00006 | M01272 | V$SOX2_Q6 |
| 2 | 0,001 | M01224 | V$P50RELAP65_Q5_01 |
| 3 | 0,002 | M00052 | V$NFKAPPAB65_01 |
| 4 | 0,002 | M01202 | V$VDRRXR_01 |
| 5 | 0,004 | M00260 | V$HLF_01 |
| **6** | **0,005** | **M00201** | **V$CEBP*** |
| 7 | 0,005 | M00054 | V$NFKAPPAB_01 |
| 8 | 0,006 | M00053 | V$CREL_01 |
| 9 | 0,006 | M00228 | V$VBP_01 |
| 10 | 0,010 | M00711 | V$ZTA_Q2 |

*The CEBPB binding motif is p < 0.00041 with an input DNA sequence that comprises the CEBPB motif +/- 5bp.

**ChIP-Seq peak 2**

Factor: CEBPB

Cluster Score (out of 1000): 248

Position: chr17:41811788-41812043

Genomic Size: 256

cell type: **IMR-90**: Cluster Score Signal (out of 1000) = 248

>hg19_dna range=chr17:41811788-41812043 5'pad=0 3'pad=0 **strand=+** repeatMasking=none

TCAGCCTTTCTGGGGCATCCCCTGCAAGATAGGCACCGGCACTGCCTGCT

CTCTCTCTCCCTGGATCTGATTTAGCCTTCACTGAAACCCTGCCCAAGAT

GATGACTTAATCTCTGGGCTGAGCGAATGCAGCCAATATGTTCAG ATGGA

TTTTGCAAGACAAAAGCTCAAATCTGTTTTGTGGCTGCAGATACACATTC

AGACTGAACCACATCCGTGGTCATTCCAGTCACAAACACAAAGGGTGCCA

TGAATT

CEBPB binding motif

GFI binding motif

This table shows extracted p-values, Matrix IDs, Matrix names, and calculated 'corrected P_values' from the DNA sequence of **ChIP-Seq peak 2**. It is sorted form the lowest to the highest p-value.

| Rank | P-value | Matrix_ID | Matrix_name |
| --- | --- | --- | --- |
| 1 | 0,00027 | M01124 | V$OCT4_02 |
| 2 | 0,004 | M00051 | V$NFKAPPAB50_01 |
| 3 | 0,006 | M01028 | V$NRSF_Q4 |
| 4 | 0,006 | M01125 | V$OCT4_01 |
| **5** | **0,007** | **M01067** | **V$GFI1_Q6** |
| 6 | 0,007 | M01590 | V$SMAD1_01 |
| **7** | **0,009** | **M00250** | **V$GFI1_01** |
| **8** | **0,009** | **M00201** | **V$CEBP_C** |
| 9 | 0,010 | M00924 | V$AP1_Q2_01 |
| 10 | 0,013 | M01272 | V$SOX2_Q6 |
| **11** | **0,0142** | **M00117** | **V$CEBPB_02*** |

*The CEBPB binding motif is p < 0.00083 with an input DNA sequence that comprises the CEBPB motif +/- 5bp.

**Distances of CEBPB motif C alleles**

**CEBPB (peak 1)**: Position C: chr17:41810515

(Location CEBPB Motif 1: 41810511 - 41810519)

**CEBPB (peak 2)**: Position C: chr17:41811942

Distance between C alleles of peak 1 and peak 2: 1427 bp

(Location CEBPB Motif 2: 41811937 – 41811946)

**dbSNP: rs9783823**: Position: chr17:41813369

Distance between C alleles of peak 2 and rs9783823: 1427 bp

**Appendix Figures**


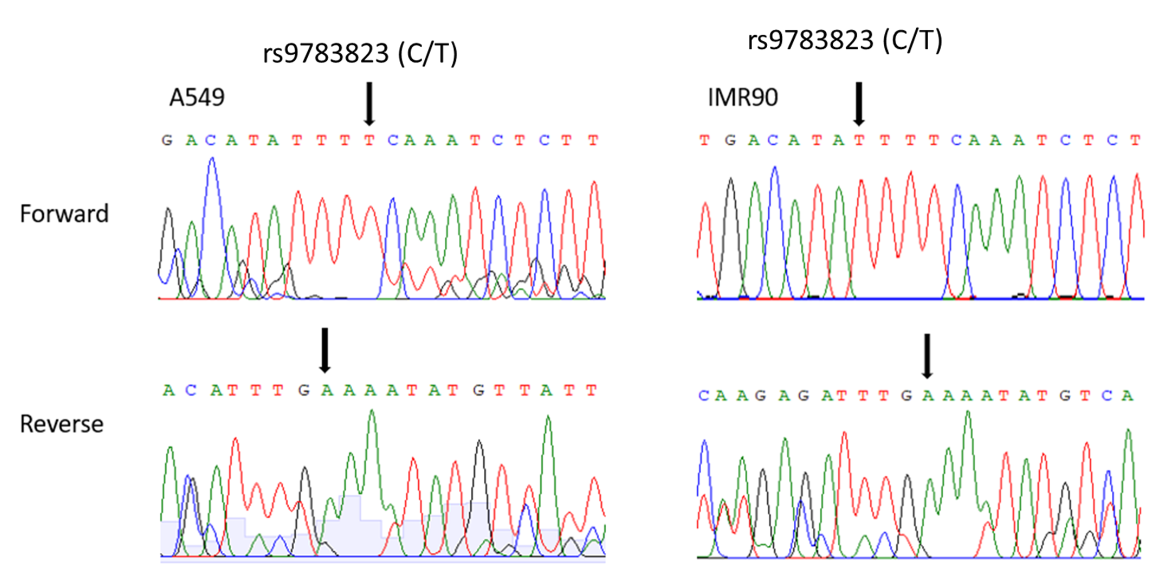


**Appendix Figure 1.** A549 and IMR90 cells are homozygous for the effect T-allele of rs9783823, which impairs the predicted CEBPB binding site.

DNA was extracted from cell pellets of A549 and IMR90 cells and PCR amplified a region up- and downstream of rs9783823. The 279 bp PCR product was sequenced in both directions. C = REF, common in AFR and predicted CEBPB binding allele, T = ALT, common in EUR.


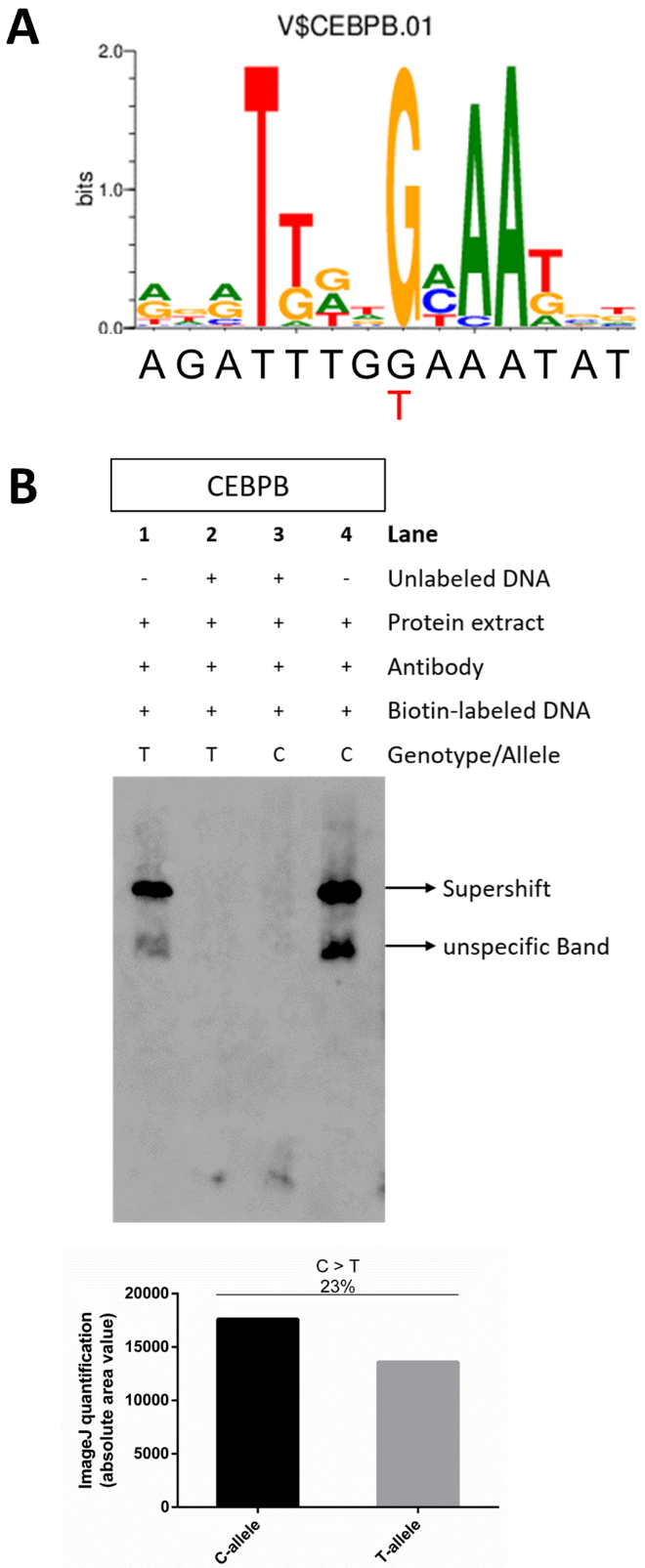


**Appendix Figure 2.** rs9783823-C allele provides a binding motif for the TF CEBPB.

(**A**). The rs9783823-T allele reduces the predicted affinity of CEBPB binding. (**B**). CEBPB binding at the SNP sequence was detected by antibody-EMSA. We observed a 23% reduction in CEBPB binding in the presence of the alternative T-allele (in the EMSA replication we observed a 14% reduction in CEBPB binding, see Figure 2).


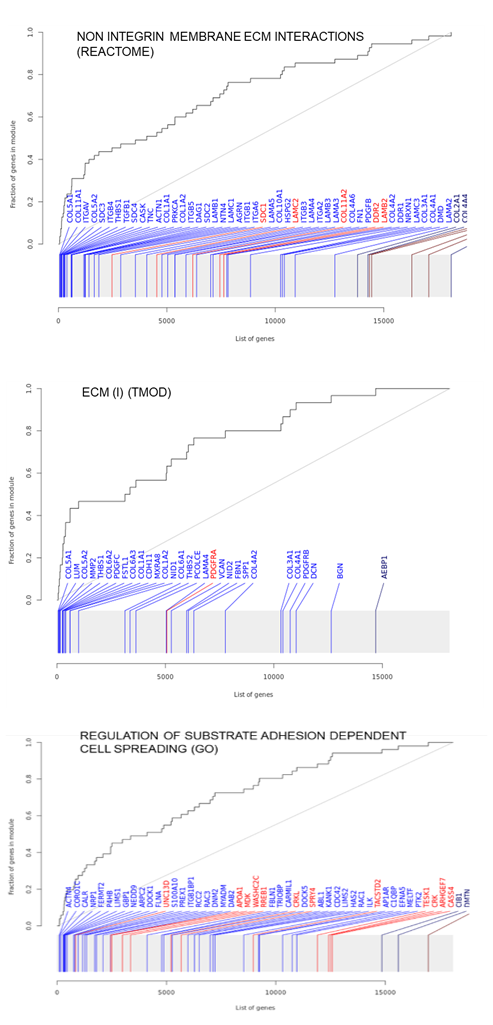


**Appendix Figure 3.** Most significantly enriched gene sets after *CEBPB* knockdown in SaOS-2 cells for 48 h from the gene set databases REACTOME, TMOD, and GO.

**Appendix Tables**

**Appendix Table 1.** SNPs in strong LD (r^2^ > 0.8) in GWAS-associated with periodontitis and bone mineral density (BMD). Gray shadow: 14 SNPs within the identified enhancer most cis-regulate *SOST* expression.

| **RS_Number** | **chr** | **hg19 position (bp)** | **distance (bp)** | **Alleles** | **MAF** | **D_prime_** | **R^2^** | **GWAS-Lead SNP of trait** | **Pubmed ID** |
| --- | --- | --- | --- | --- | --- | --- | --- | --- | --- |
| rs8080687 | chr17 | 41803034 | 0,049 | (A/G) | 0.3658 | 1.0 | 0.9775 |  |  |
| rs8073524 | chr17 | 41803083 | 1,381 | (C/G) | 0.3632 | 1.0 | 0.9887 |  |  |
| **rs6416905** | chr17 | 41804464 | 2,507 | (A/G) | 0.3605 | 1.0 | 1.0 | **periodontitis** | **31537151** |
| rs6503474 | chr17 | 41806971 | 0,011 | (A/G) | 0.3632 | 1.0 | 0.9887 |  |  |
| rs9913749 | chr17 | 41806982 | 0,328 | (A/G) | 0.3658 | 1.0 | 0.9775 |  |  |
| rs1513671 | chr17 | 41807310 | 0,021 | (G/C) | 0.3605 | 0.9886 | 0.9773 |  |  |
| **rs1513670** | chr17 | 41807331 | 0,39 | (T/C) | 0.3605 | 0.9886 | 0.9773 | **BMD** | **26733130, 19079262** |
| rs1513669 | chr17 | 41807721 | 0,882 | (A/G) | 0.3632 | 1.0 | 0.9887 |  |  |
| rs34635683 | chr17 | 41808603 | 0,507 | (G/-) | 0.3632 | 1.0 | 0.9887 |  |  |
| rs34423381 | chr17 | 41809110 | 0,158 | (T/-) | 0.3947 | 0.9638 | 0.8031 |  |  |
| rs1976960 | chr17 | 41809268 | 0,579 | (T/C) | 0.3632 | 1.0 | 0.9887 |  |  |
| rs1534402 | chr17 | 41809847 | 0,277 | (C/T) | 0.3632 | 1.0 | 0.9887 |  |  |
| rs1534401 | chr17 | 41810124 | 0,189 | (C/T) | 0.3632 | 1.0 | 0.9887 |  |  |
| rs8071941 | chr17 | 41810313 | 0,261 | (G/A) | 0.3632 | 1.0 | 0.9887 |  |  |
| rs1969505 | chr17 | 41810574 | 0,434 | (T/C) | 0.3632 | 1.0 | 0.9887 |  |  |
| rs6503475 | chr17 | 41811008 | 0,148 | (G/A) | 0.3632 | 1.0 | 0.9887 |  |  |
| rs11279297 | chr17 | 41811156 | 1,405 | (CTCCAG/-) | 0.3632 | 1.0 | 0.9887 |  |  |
| rs955412 | chr17 | 41812561 | 0,808 | (T/C) | 0.3632 | 1.0 | 0.9887 |  |  |
| **rs9783823** | chr17 | 41813369 | 0,26 | (C/T) | 0.4132 | 1.0 | 0.8008 | **causal variant** |  |
| rs9783806 | chr17 | 41813629 | 0,28 | (T/G) | 0.4132 | 1.0 | 0.8008 |  |  |
| rs9902196 | chr17 | 41813909 | 0,063 | (C/T) | 0.4132 | 1.0 | 0.8008 |  |  |
| rs36193607 | chr17 | 41813972 | 0,036 | (T/-) | 0.3632 | 1.0 | 0.9887 |  |  |
| rs9303537 | chr17 | 41814008 | 0,075 | (T/G) | 0.3632 | 1.0 | 0.9887 |  |  |
| rs9303538 | chr17 | 41814083 | 0,893 | (G/A) | 0.4132 | 1.0 | 0.8008 |  |  |
| rs9915878 | chr17 | 41814976 | 0,79 | (G/A) | 0.4132 | 1.0 | 0.8008 |  |  |
| rs9303539 | chr17 | 41815766 | 1,386 | (G/A) | 0.4132 | 1.0 | 0.8008 |  |  |
| rs2076793 | chr17 | 41817152 | 0,359 | (A/T) | 0.3605 | 0.9886 | 0.9773 |  |  |
| rs9908933 | chr17 | 41817511 | 0,119 | (T/C) | 0.3605 | 0.9886 | 0.9773 |  |  |
| rs9303540 | chr17 | 41817630 | 18,993 | (T/C) | 0.3579 | 0.9885 | 0.966 |  |  |
| rs851054 | chr17 | 41836623 | 0,237 | (C/T) | 0.3526 | 0.9533 | 0.8781 |  |  |
| rs851055 | chr17 | 41836860 | 0,322 | (G/A) | 0.3526 | 0.9533 | 0.8781 |  |  |
| rs851056 | chr17 | 41837182 | 0,323 | (G/C) | 0.3526 | 0.9533 | 0.8781 |  |  |
| rs200083764 | chr17 | 41837505 | 0,724 | (TCC/-) | 0.3526 | 0.9533 | 0.8781 |  |  |
| rs1237278 | chr17 | 41838229 | 0,665 | (C/T) | 0.3553 | 0.9537 | 0.8889 |  |  |
| rs1708635 | chr17 | 41838894 | 0,175 | (A/T) | 0.3553 | 0.9537 | 0.8889 |  |  |
| rs1634330 | chr17 | 41839069 | -36,591 | (T/C) | 0.3553 | 0.9537 | 0.8889 |  |  |

**Appendix Table 2.** Oligonucleotides of the CEBPB and GFI-1 EMSA probes.

| **Probe**  **length: 43 bp** | **Forward (5'-3')** | **Reverse (5'-3')** | **3' Modification** |
| --- | --- | --- | --- |
| CEBPB_  rs9783823-C | AGTTTTTGTACTGACATATTTCCAAATCTCTTGGGTATATACC | GGTATATACCCAAGAGATTTGGAAATATGTCAGTACAAAAACT | Biotin |
|  |  |  |  |
| CEBPB_  rs9783823-C | AGTTTTTGTACTGACATATTTCCAAATCTCTTGGGTATATACC | GGTATATACCCAAGAGATTTGGAAATATGTCAGTACAAAAACT | - |
|  |  |  |  |
| CEBPB_  rs9783823-T | AGTTTTTGTACTGACATATTTTCAAATCTCTTGGGTATATACC | GGTATATACCCAAGAGATTTGAAAATATGTCAGTACAAAAACT | Biotin |
|  |  |  |  |
| CEBPB_  rs9783823-T | AGTTTTTGTACTGACATATTTTCAAATCTCTTGGGTATATACC | GGTATATACCCAAGAGATTTGAAAATATGTCAGTACAAAAACT | - |
|  |  |  |  |
| GFI-1_  rs8071941-G | GGAGGCAATTCCAGACAAAGGGATCAGTGCAAGCAAAGGAAGC | GCTTCCTTTGCTTGCACTGATCCCTTTGTCTGGAATTGCCTCC | Biotin |
|  |  |  |  |
| GFI-1_  rs8071941-G | GGAGGCAATTCCAGACAAAGGGATCAGTGCAAGCAAAGGAAGC | GCTTCCTTTGCTTGCACTGATCCCTTTGTCTGGAATTGCCTCC | - |
|  |  |  |  |
| GFI-1_  rs8071941-A | GGAGGCAATTCCAGACAAAGGAATCAGTGCAAGCAAAGGAAGC | GCTTCCTTTGCTTGCACTGATTCCTTTGTCTGGAATTGCCTCC | Biotin |
|  |  |  |  |
| GFI-1_  rs8071941-A | GGAGGCAATTCCAGACAAAGGAATCAGTGCAAGCAAAGGAAGC | GCTTCCTTTGCTTGCACTGATTCCTTTGTCTGGAATTGCCTCC | - |

**Appendix Table 3.** Cloning Primers used for Reporter Gene Assays.

| **Probe**  **construct length w/o restriction sites** | **Forward (5'-3')** | **Reverse (5'-3')** | **Description** |
| --- | --- | --- | --- |
| PCR_HindIII_  near to rs9783823  *78 bp in CEBPB* | CCCAAGCTTTATGAACATTCATGTGC | CCCAAGCTTACGTCAATTCCACTCC | tagged by rs9783823 |
| PCR_HindIII_  near to rs8071941  *190 bp in GFI-1* | CCCAAGCTTATATCTACACGAAGGATGGGTAC | CCCAAGCTTAACAAATGCCAGCCTATCTTCA | tagged by rs8071941 |
| PCR_pGL4.24_  Backbone  *360 bp* | AGAGCCTTCAACCCAGTCAG | GTTTCGCCACCTCTGACTTG | pGL4.24 Backbone |

**Appendix Table 4.** Oligonucleotides of the CRISPRa *SOST* sgRNA probes.

| **gRNA Probe** | **Target SNP** | **Coord** | **hg19 location (bp)** | **distance (bp) to SNP** | **Forward (5'-3')** | **Reverse (5'-3')** |
| --- | --- | --- | --- | --- | --- | --- |
| gRNA_  rs851055 | rs851055 | chr17 | 41836820 | -40 | CAC CGT TAC AAA TCG AGG ATG CGC | AAA CGC GCA TCC TCG ATT TGT AAC |
| gRNA_  rs851055_2 | rs851055 | chr17 | 41836767 | -93 | CAC CGA ACT AGA GGG TCA GAG CAA | AAA CTT GCT CTG ACC CTC TAG TTC |
| gRNA_  rs851056 | rs851056 | chr17 | 41837216 | 34 | CAC CGC TCA TTA TAT GCA GGG CCA | AAA CTG GCC CTG CAT ATA ATG AGC |
| gRNA_  rs1634330 | rs1634330 | chr17 | 41839055 | -14 | CAC CGG TTA AAC CAC GGG AGA TGT | AAA CAC ATC TCC CGT GGT TTA ACC |
| gRNA_  rs9303540 | rs9303540 | chr17 | 41817601 | -29 | CAC CGC TCC TCA GGA GAC CCG CGT | AAA CAC GCG GGT CTC CTG AGG AGC |
| gRNA_  rs35174738 | rs35174738 | chr17 | 41817360 | -64 | CAC CGT GGC TCG GGA CCC CAG GAG | AAA CCT CCT GGG GTC CCG AGC CAC |
| gRNA_  rs9303539 | rs9303539 | chr17 | 41815917 | 151 | CAC CGC CCC CTT GGC TGG TAC CAG | AAA CCT GGT ACC AGC CAA GGG GGC |
| gRNA_  rs9915878 | rs9915878 | chr17 | 41814704 | -272 | CAC CGC TCT AGC TGG GGC CGG GAT | AAA CAT CCC GGC CCC AGC TAG AGC |
| gRNA_  rs8071941 | rs8071941 | chr17 | 41810255 or 41810358 | -58 or 45 | CAC CGG ACA GTT ATA TCT ACA CGA | AAA CTC GTG TAG ATA TAA CTG TCC |
| gRNA_  rs1969505 | rs1969505 | chr17 | 41810537 | -37 | CAC CGT TAT TCA TAG TGA GCC ACT | AAA CAG TGG CTC ACT ATG AAT AAC |
| gRNA_  rs6503475 | rs6503475 | chr17 | 41811053 | 45 | CAC CGA TCA GAA ATG CAG GTA GCC | AAA CGG CTA CCT GCA TTT CTG ATC |
| gRNA_  rs9783806 | rs9783806 | chr17 | 41813531 | -98 | CAC CGT TTA CTG CCC CTT ACT CTA | AAA CTA GAG TAA GGG GCA GTA AAC |
| gRNA_  rs1513671 &rs1513670 | rs1513671  rs1513670 | chr17  chr17 | 41807357 or 41807206 | 47 or -104  26 or -125 | CAC CGC CGT CCT GCA AGA TCC AGA | AAA CTC TGG ATC TTG CAG GAC GGC |

**Appendix Table 5.** TRAP predicted the highest allele-specific effects on TF CEBPB for rs9783823.

| **allele** | **lost/**  **new** | **Family/**  **matrix** | **Further Information** | **Opt. thresh.** | **Start pos.** | **End pos.** | **Core sim.** | **Matrix sim.** |
| --- | --- | --- | --- | --- | --- | --- | --- | --- |
| C -> T | lost | V$NFAT/NFAT5.02 | Nuclear factor of activated T-cells 5 | 0,87 | 38 | 56 | 1 | 0,903 |
| C -> T | lost | V$NFAT/NFAT.01 | Nuclear factor of activated T-cells | 0,95 | 40 | 58 | 1 | 0,95 |
| **C -> T** | **lost** | **V$CEBP/CEBPB.01** | **CCAAT/enhancer binding protein beta** | **0,94** | **45** | **59** | **1** | **0,979** |
| C -> T | new | V$CHRF/CHR.01 | Cell cycle gene homology region (CDE/CHR tandem elements regulate cell cycle dependent repression) | 0,92 | 46 | 58 | 1 | 0,928 |
| C -> T | new | V$GATA/GATA3.02 | GATA-binding factor 3 | 0,91 | 49 | 61 | 1 | 0,937 |
